# The outcomes and complications of percutaneous interventions in chronic total coronary occlusion

**DOI:** 10.1186/s43044-024-00490-6

**Published:** 2024-05-23

**Authors:** Muhammad Suleman, Nayyar Arif, Muhammad Ishaq Khan, Muhammad Saad Jibran, Muhammad Jamil, Shafi Ullah Khan, Shah Sawar Khan, Ghulam Rasool Maken

**Affiliations:** 1 Department of Cardiology, Armed Forces Institute of Cardiology, Rawalpindi, Punjab Pakistan; 2 Department of Cardiology, Peshawar Institute of Cardiology, Peshawar, Khyber Pakhtunkhwa Pakistan; 3 Department of Cardiology, Mufti Mehmood Memorial Teaching Hospital, Dera Ismail Khan, Khyber Pakhtunkhwa Pakistan; 4 Department of Cardiology, Services Hospital Peshawar, Peshawar, Pakistan; 5 Department of Medicine, District Headquarters Teaching Hospital, Kohat, Khyber Pakhtunkhwa Pakistan

**Keywords:** Coronary occlusion, Percutaneous coronary intervention, Coronary angiography

## Abstract

**Background:**

The limited availability of complex coronary intervention facilities and qualified operators, due to the high cost associated with chronic total occlusion (CTO) percutaneous intervention (PCI) equipment and a shortage of necessary skills, has led to a scarcity of capable medical centers in Pakistan. This study seeks to examine the outcomes and potential complications associated with CTO PCI procedures conducted at the Cardiac Catheterization Laboratories of a prominent national institute in Pakistan, which handles a large volume of cases.

**Results:**

Three hundred and six patients were included in the study in the study period of six months. The mean age was 59.49 (± 9.16) years: 256 (83.66%) were male and 50 (16.34%) were female. CTO was successfully re-vascularized in 237 (77.5%) with a complication rate of 13.7%. Two hundred and ninety-eight (97.39%) patients underwent an antegrade approach, while RCA was the most common target vessel (47.71%). Diabetes was the only significant associated risk factor with CTO PCI failure (30.43% vs. 30.43%, *P*-value = 0.015).

**Conclusion:**

We achieved an excellent procedural success rate with a low complication rate. CTO procedural failure is associated with a higher complication rate, and diabetes is among the risk factors that lead to higher procedural failure.

## Background

Cardiovascular disease (CVD) is the most widespread cause of mortality globally. Global health projections predict CVD to remain the foremost cause of mortality in 2030 [1]. Chronic total occlusions (CTOs) of coronary arteries are prevalent in 15–30% of patients undergoing coronary angiography [2]. CTO lesions often result in ischemia, and research indicates that the usual coronary flow reserve is observed in less than 10% of patients despite collateral circulation [3].

Recent studies have indicated that chronic total occlusion (CTO) positively correlates with a high mortality rate [4]. Additionally, it has been established that CTO significantly contributes to coronary artery bypass graft (CABG) surgery referrals [5]. Successful revascularization of CTO has significantly improved symptoms, left ventricular function, a reduction in arrhythmias, and better tolerance of acute coronary syndrome [6]. In contrast, failure to revascularize CTO has been linked to an increased risk of mortality and angina pectoris compared to successful revascularization in meta-analyses [6, 7].

Despite the high prevalence of chronic total occlusions (CTO) encountered during coronary angiography procedures, the technical challenges and inadequate procedural success rates have resulted in a comparatively lower reported incidence of CTO percutaneous coronary intervention (PCI), ranging from 3.8 to 4.8% of total PCI volumes in the National Cardiovascular Data Registry in the USA [8]. A Pan-London CTO cohort study has reported a procedural success rate of 76.9%, with a 4.2% complication rate in the procedural success group and a 16.2% complication rate in the procedural failure group [9]. The non-affordability of CTO PCI equipment for patients and the lack of requisite skills among operators have resulted in a limited number of centers in Pakistan capable of performing complex coronary interventions. Consequently, the rate of procedural success and the incidence of complications may be lower than those reported in developed nations, necessitating the reporting of procedural outcomes and complications in our national cardiac institutes.

This study aims to investigate the outcomes and complications of patients who undergo chronic total occlusion (CTO) percutaneous coronary intervention (PCI) at the Cardiac Catheterization Laboratories of a high-volume national institute.

## Methods

### Study design

Descriptive case series.

### Study setting

Cardiac Catheterization laboratories at the Armed Forces Institute of Cardiology.

### Study duration

Six months from 31 August 2019 to 29 February 2020.

### Sample size

The sample size was 273 patients, calculated assuming a confidence interval of 95% using the study from Jones DA et al. [9], who reported procedural success of CTO PCI at 76.9%**.**

**Table:** 

Hypothesized % frequency of outcome factor in the population (p):	76.9% ± 5
Confidence limits as % of 100(absolute ± %)(d):	5%
Design effect (for cluster surveys-DEFF):	1
$${\text{Sample size }}n \, = \, \left[ {{\text{DEFF}}*{\text{Np }}\left( {1 - p} \right)} \right]/\left[ {d^{2} /Z^{2}_{1 - \alpha /2} *\left( {N - 1} \right) \, + p*\left( {1 - p} \right)} \right]$$	
Confidence level 95%	
Sample size 273	

### Sampling technique

Non-randomized consecutive sample.

### Sample selection

#### Inclusion criteria

We enrolled patients of both genders with ages more than 35 years who had angina not responding to medical therapy and underwent antegrade or retrograde CTO PCI at AFIC/NIHD for revascularization during the study period.

#### Exclusion criteria

To control confounding bias

- Patients who had myocardial infarction of the target vessel for which CTO PCI is planned within the last three months
- Patients who underwent PCI of some other lesion in the same index procedure are excluded from the study.

### Data collection

A non-randomized consecutive sample consisting of 306 patients who underwent chronic total occlusion (CTO) percutaneous coronary intervention (PCI) at the Armed Forces Institute of Cardiology & National Institute of Heart Diseases (AFIC & NIHD) was selected based on their eligibility criteria. The ethical board of our hospital gave approval for patient data and advised us to forego the formal approval process as the patients underwent a routine procedure in the hospital. To minimize confounding bias, patients who did not meet the inclusion criteria were excluded from the study. The patients were informed about the study and provided with their consent. Before the procedure, a detailed medical history and physical examination were conducted. A resident cardiologist completed the biodata of the patients and the pre-procedural section. The outcome variable of procedural success was documented in the catheterization laboratory after the procedure was completed. In contrast, procedural complications were recorded during the procedure at the catheterization laboratory. Major adverse cardiac events (MACEs) were documented during the hospital stay.

### Data analysis

In descriptive statistics, categorical variables such as gender, diabetes, hypertension, angina class, dyspnea NYHA class, target vessel, TIMI flow, and history of MI were presented as frequencies. Meanwhile, continuous variables, including age, ejection fraction, number of vessels, number of previous attempts, number of stents, number of wires, and number of balloons, were presented as means with their corresponding standard deviations.

The outcome variable, procedural success, failure, and complication, was also presented as frequencies. To compare the occurrence of complications between the groups that experienced procedural success and failure, a Pearson Chi-square test was conducted for parametric data. Effect modifiers, such as age, gender, hypertension, diabetes, and dyslipidemia, were controlled by stratification. Post-stratification Chi-square test was then applied. The statistical significance was established at a *p*-value of < 0.05 when comparing means.

## Results

Our study included 306 patients with CTO who met our inclusion and exclusion criteria. Most of the patients were male (83%, *n* = 256), with a mean age of 59.49 ± 9.16. The age group most affected was 61 to 75 years (42.16%, *n* = 129). Of the enrolled patients, 24.18% (*n* = 74) had hypertension, 19.61% (*n* = 60) had diabetes mellitus, 43.46% (*n* = 133) had dyslipidemia, and 66.99% (*n* = 205) had a history of myocardial infarction. Among the patients, 230 (75.14%) had angina, with the majority reporting class IV (27.12%, *n* = 83). Dyspnea was reported by 230 patients (75.16%), with NYHA class III being the most commonly reported (26.80%, *n* = 82). Table 1 presents the baseline characteristics.

**Table 1:** Baseline characteristics of study subjects (*n* = 306)

Variable		Mean ± SD	Frequency	Percentage
Age		59.49 ± 9.16		
Hypertension		–	74	24.18%
Male gender		–	256	83.66%
Diabetes mellitus		–	60	19.61%
Dyslipidemia		–	133	43.46%
History of myocardial infarction		–	205	66.99%
History of PCI			19	5.5%
History of CABG			6	1.96%
Ejection fraction		42.43 (± 11.10)		
Hemoglobin		12.14 (± 1.94)		
Platelet		172.47 (± 42.91)		
WBC		7313.73 (± 2174.28)		
Creatinine		0.97 (± 0.31)		
Angina CCS class	Class I	–	75	24.51%
	Class II	–	79	25.82%
	Class III	–	69	22.55%
	Class IV	–	83	27.12%
Dyspnea NYHA class	None	–	76	24.84%
	NYHA 1	–	76	24.84%
	NYHA II	–	72	23.53%
	NYHA III	–	82	26.80%
	NYHA IV	–	0	0%

The procedural characteristics of our patients are presented in Table 2. The majority of patients had single vessel disease (*n* = 110, 35.95%), with the right coronary artery (RCA) being the most frequently affected target vessel (*n* = 146, 47.71%). The antegrade approach was the most commonly used intervention method for PCI (*n* = 298, 97.39%), while only eight patients underwent the retrograde approach.

**Table 2:** Procedural characteristics

Variable		Mean +-SD	Frequency	Percentage
No. of vessels involved	SVCAD	–	110	35.95%
	DVCAD	–	93	30.39%
	TVCAD	–	103	33.66%
Approaches	Antegrade	–	298	97.39%
	Retrograde	–	8	2.61%
	ADR	–	0	0%
Target vessel	RCA	–	146	47.71%
	LAD	–	115	37.58%
	LCX	–	45	14.71%
Contrast volume		269.54 (± 115.86)		
Stents		1.82 (± 1.27)		
Wires		3.48 (± 1.90)		
Ballon		4.42(± 2.81)		
Catheters		1.92(± 1.15)		

The outcome and complication details of our patients are presented in Table 3. The data indicate an overall success rate of 77.45% (*n* = 237), while complications were observed in 42 patients (13.73%). The most commonly reported complication was vessel dissection, accounting for 23 cases (54.76%).

**Table 3:** Outcomes and complications

		Frequency	Percentage (%)
CTO outcome	Failure	69	22.55
	Success	237	77.45
CTO complications	All	42	13.73
	Dissection	23	7.52
	Perforation	8	2.61
	MACE	9	2.94
	No reflow	2	0.65

Table 3 displays how variables are stratified according to the outcome of CTO (chronic total occlusion) treatment. Among the 237 successful cases, patients over 75 years old (*n* = 12, 100%), females (*n* = 40, 80%), those with class 3 angina (*n* = 55, 79.71%), NYHA class III dyspnea (n = 66, 80.49%), triple vessel disease (*n* = 81, 78.64%), retrograde approach (*n* = 8, 87.5%), left circumflex artery (*n* = 37, 82.22%). Higher operator volume (84.4%, *n* = 130) had the highest success rate. Diabetes and operator volume were the only variables significantly associated with CTO outcome.

Perforation (75%, *n* = 6) and dissection (60.87%, *n* = 14) were the complications with the highest failure rate. The failure group had a higher complication rate compared to the successful group, and this difference was statistically significant. A detailed stratification of all variables against the success and failure of PCI (percutaneous coronary intervention) in CTO patients is presented in Table 4. A *p*-value below 0.05 indicates a significant association.

**Table 4:** Stratification of variables against CTO outcomes (*n* = 306)

Variables		CTO outcome			*P* valve
		Success (*n* = 237)	Failure (*n* = 69)	Total	
Age	Below 50	39 (75%)	13 (25%)	52 (16.99%)	0.293
	51–60	87 (76.99%)	26 (23.01%)	113 (36.93%)	
	61–75	99 (76.74%)	30 (23.26%)	129 (42.16%)	
	Above 75	12 (100%)	0 (0%)	12 (3.92%)	
Gender	Male	197 (76.95%)	59 (23.04%)	256 (83.66%)	0.714
	Female	40 (80%)	10 (20%)	50 (16.34%)	
DM		39 (65%)	12 (%)	60 (19.60%)	0.015
HTN		59 (79.73%)	15 (20.27%)	74 (24.18%)	0.635
Smoker		28 (82.35%)	6 (17.65%)	34 (11.11%)	0.460
Dyslipidemia		101 (75.94%)	32 (24.06%)	133 (43.46%)	0.584
Angina	Class 1 (*n* =	57 (76%)	18 (24%)	75 (24.51%)	0.96
	Class 2 (*n* =	61 (77.22%)	18 (22.78%)	79 (25.82%)	
	Class 3 (*n* =	55 (79.71%)	14 (20.29%)	69 (22.55%)	
	Class 4 (*n* =	64 (77.11%)	19 (22.89%)	83 (27.12%)	
Dyspnea	None	62 (79.49%)	14 (20.51%)	76 (24.84%)	0.46
	Class I	57 (75%)	19 (25%)	76 (24.84%)	
	Class II	52 (72.22%)	20 (27.78%%)	72 (23.53%)	
	Class III	66 (80.49%)	16 (19.51%)	82 (26.80%)	
	Class IV	0	0	0	
History of MI		161 (78.54%)	44 (21.46%)	205 (66.99%)	0.561
History of NSTEMI		24 (80%)	6 (20%)	30 (9.80%)	0.715
History of PCI		19 (73.08%)	7 (26.92%)	26 (8.50%)	0.587
History of CABG		6 (100%)	0	6 (1.96%)	0.181
ETT		9 (64.29%)	5 (35.71%)	14 (4.58%)	0.232
Inferior MI		18 (90%)	2 (10%)	20 (6.54%)	0.162
Anterior MI		30 (81.08%)	7 (18.92%)	37 (12.09%)	0.563
Number of vessels	SVCAD	85 (77.27%)	25 (22.73%)	110 (35.95%)	0.93
	DVCAD	71 (76.34%)	22 (23.66%)	93 (30.39%)	
	TVCAD	81 (78.64%)	22 (21.36%)	103 (33.66%)	
Approach	Antegrade	230 (77.18%)	68 (22.82%)	298 (97.39%)	0.69
	Retrograde	7 (87.5%)	1 (12.5%)	8 (2.61%)	
Complications (*n* = 42)	Dissection	9 (39.13%)	14 (60.87%)	23 (7.52%)	< 0.0001
	Perforation	2 (25%)	6 (75%)	8 (2.61%)	
	MACE	4 (44.44%)	5 (55.56%)	9 (2.94%)	
	No reflow	0 (0%)	2 (100%)	2 (0.65%)	
	Missing	215 (85.32%)	37 (14.68%)	252 (82.35%)	
Target vessels	RCA	107 (73.29%)	39 (26.71%)	146 (47.71%)	0.25
	LAD	93 (80.87%)	22 (19.13%)	115 (37.58%)	
	LCX	37 (82.22%)	8 (17.78%)	45 (14.71%)	
Operator volume	Low volume	12 (63.2%)	7 (36.8%)	19 (6.2%)	0.010
	Medium volume	95 (71.4%)	38 (28.6%)	133 (43.5%)	
	High volume	130 (84.41%)	24 (15.59%)	154 (50.3%)	
Ejection fraction		42.59 (± 10.96)	41.88 (± 11.63)	42.43 (± 11.10)	0.39

## Discussion

This study at our center had a high procedural success of 77.4% despite a very low retrograde approach (2.64%). It was as high as 84.1% when performed by operators with high-volume experience and as low as 63.2% with low-volume operators. The success rates are slightly lower than those reported in international registries and meta-analysis studies worldwide [10, 11]. These stats can improve further if a proper retrograde PCI program and dedicated PCI CTO fellowship training become available in our country. Studies have shown that initiating CTO PCI fellowship training programs at 1-year training can give success rates similar to those of high-volume experienced PCI centers [12].

Although the success rate of retrograde PCI is better, its complications rate and MACE are reported to be 4.3% compared to 1.1% of the antegrade approach, which was reduced to 3% using hybrid algorithms [13]. This could be because this advanced procedure is preferred for complicated cases like calcified vessels and larger lesions requiring higher procedure time, operator expertise, contrast volume, and radiation. The complication rate could be reduced further by preferring septal collaterals over epicardial collateral in the retrograde approach, as reported by the Japanese registry, where MACE was reduced to 1.5% [14]. The progress CTO registry reports a mean procedural success of 87.9% [15].

Our study endorses the safety of PCI in CTO outcomes with a minimum complication rate of 13.73%, dissection of 7.52%, coronary artery perforation of 2.61%, and in-hospital MACE of 2.94%(*n* = 9). The coronary perforation rate in our CTO patients is significantly lower (2.61%, *n* = 8) than the internationally reported perforation rate of 8.8% in the Open CTO registry [16]. The most common cause of procedural failure was a failure to cross the wire (41.42%), followed by failure to cross the balloon (32.86%). Failure due to complications was reported in 25.71% of cases. These complications and failure rates are coherent with international data and even better in some parameters [10, 17, 18].

Some studies in the literature analyzing Progress CTO Registry showed increased failure rates and MACE in CTO interventions with advancing age [5]. CTO progress registry showed a sevenfold increased complication rate in ages above 75 and a fourfold increase when older than 65 compared to patients younger than 65 [19]. In contrast, others found no correlation between age and procedural success and showed better survival outcomes in old ages compared to younger patients [20]. Our data were coherent with later studies and had a 100% success rate in age groups above 75 (*n* = 12). This could be due to our selection bias for cases above 75 yrs age, where the less complex disease was chosen for revascularization, and very complex diseases were not.

Contrary to international data that report lower success rates in CTO involving left circumflex artery [21], our study had a higher success rate of 82.22%(*n* = 37) compared to other vessels (LAD 80.87% *n* = 93, and RCA 73.29% *n* = 107).

A meta-analysis compared successful PCI in CTO vs failed PCI for 3.1 years of mean follow-up and found that successful PCI had the better mean procedural success of 71% (51–87% range), fewer angina symptoms (OR 0.38 CI 95%,0.24–0.60), lower stroke risks (OR 0.72, CI 95%, 0.60–0.88), lower MACE risk (OR 0.59, CI 95%, 0.44–0.79), reduced need to follow up CABG (OR 0.18, CI 95%,0.14–0.22), and reduced risk of mortality (OR 0.52, CI 95%, 0.43–0.63) [22].

The contrast volume required during the CTO procedure was 250 ml. The median number of wires required was three, while four balloons were used per procedure. Antegrade wire escalation requires a minimum of two wires, one workhorse for reaching the proximal cap of the lesion and the second special CTO wire. Similarly, more balloons are required for pre-dilatation of the CTO lesion as smaller balloons are usually required to cross the tightly stenotic CTO. More catheters were used in failed CTO PCI (2 vs. 1), perhaps to increase the support for crossing the lesion with wire. All these protocols were consistent with multicenter US registries [23, 24].

In terms of limitation, this was a single-center non-randomized observational study with no long-term follow-up. Our study did not report lesion length, complexity, or risk prediction scores like JCTO. Only in-hospital MACE outcome was reported. Patient selection criteria were not reported, and all patients were included in the specific period. Low-volume operators were included, which could have reduced the success rate. We suggest randomized controlled trials throughout the country with multicenters to assess the actual procedural success rates and procedure complication rates and to gauge better the risk–benefit ratio of CTO PCI on mortality and morbidity in such patients. As international studies report similarities, long-term outcomes of these CTO PCI must be observed and correlated with non-CTO PCI outcomes in our country [25].

## Conclusions

Our research has demonstrated a high rate of procedural success and a lower rate of complications, which aligns with findings from numerous international studies. Additionally, our study revealed that diabetes is a risk factor for CTO procedural failure associated with a higher complication rate. Furthermore, our findings suggest that a successful CTO program can be implemented in tertiary care centers in Pakistan. Moreover, the equipment and resources needed for CTO PCI are well-established and can be obtained on average.

## Data Availability

The datasets used and/or analyzed during the current study are available from the corresponding author upon reasonable request.

## Abbreviations

PCI: Percutaneous coronary Intervention
CTO: Chronic total occlusion
CABG: Coronary artery bypass grafting
LMCAD: Left main coronary artery disease
MACE: Major adverse cardiovascular event
CVD: Cardiovascular disease
CHD: Coronary heart disease
HDL: High-density lipoproteins
LDL: Low-density lipoproteins
cTn: Cardiac Troponin
ECG: Electrocardiogram
NYHA: New York Heart Association
Hb: Hemoglobin
WBC: White blood cells
DES: Drug-eluting stent
CCS: Canadian cardiovascular society
